# Comparison of symptomatic and asymptomatic atherosclerotic carotid plaques using parallel imaging and 3 T black-blood in vivo CMR

**DOI:** 10.1186/1532-429X-15-44

**Published:** 2013-05-24

**Authors:** Jochen M Grimm, Andreas Schindler, Tobias Freilinger, Clemens C Cyran, Fabian Bamberg, Chun Yuan, Maximilian F Reiser, Martin Dichgans, Caroline Freilinger, Konstantin Nikolaou, Tobias Saam

**Affiliations:** 1 Institute for Clinical Radiology, Ludwig-Maximilians-University Hospital, Munich, Germany; 2 Institute for Stroke and Dementia Research, Ludwig-Maximilians-UniversityHospital, Munich, Germany; 3 Department of Neurology, Ludwig-Maximilians-University Hospital, Munich, Germany; 4 Department of Radiology, University of WashingtonSchool of Medicine, Seattle, USA; 5 Institute of Clinical Radiology, University of Munich, Pettenkoferstr 8a, Munich, 80336, Germany

**Keywords:** Plaque imaging, Cardiovascular MR, Ischemic stroke, Vulnerable plaque, Atherosclerosis

## Abstract

**Background:**

To determine if black-blood 3 T cardiovascular magnetic resonance (bb-CMR) can depict differences between symptomatic and asymptomatic carotid atherosclerotic plaques in acute ischemic stroke patients.

**Methods:**

In this prospective monocentric observational study 34 patients (24 males; 70 ±9.3 years) with symptomatic carotid disease defined as ischemic brain lesions in one internal carotid artery territory on diffusion weighted images underwent a carotid bb-CMR at 3 T with fat-saturated pre- and post-contrast T1w-, PDw-, T2w- and TOF images using surface coils and Parallel Imaging techniques (PAT factor = 2) within 10 days after symptom onset. All patients underwent extensive clinical workup (lab, brain MR, duplex sonography, 24-hour ECG, transesophageal echocardiography) to exclude other causes of ischemic stroke. Prevalence of American Heart Association lesion type VI (AHA-LT6), status of the fibrous cap, presence of hemorrhage/thrombus and area measurements of calcification, necrotic core and hemorrhage were determined in both carotid arteries in consensus by two reviewers who were blinded to clinical information. McNemar and Wilcoxon's signed rank tests were use for statistical comparison. A p-value <0.05 was considered statistically significant.

**Results:**

Symptomatic plaques showed a higher prevalence of AHA-LT6 (67.7% vs. 11.8%; p < 0.001; odds ratio = 12.5), ruptured fibrous caps (44.1% vs. 2.9%; p < 0.001; odds ratio = 15.0), juxtaluminal thrombus (26.5 vs. 0%; p < 0.01; odds ratio = 7.3) and intraplaque hemorrhage (58.6% vs. 11.8%; p = 0.01; odds ratio = 3.8). Necrotic core and hemorrhage areas were greater in symptomatic plaques (14.1 mm^2^ vs. 5.5 mm^2^ and 13.6 mm^2^ vs. 5.3 mm^2^; p < 0.01, respectively).

**Conclusion:**

3 T bb-CMR is able to differentiate between symptomatic and asymptomatic carotid plaques, demonstrating the potential of bb-CMR to differentiate between stable and vulnerable lesions and ultimately to identify patients with low versus high risk for cardiovascular complications. Best predictors of the symptomatic side were a ruptured fibrous cap, AHA-LT 6, juxtaluminal hemorrhage/thrombus, and intraplaque hemorrhage.

## Background

Atherosclerotic disease and its complications are the leading cause of death in the industrial world [1,2]. Increasing effort has been put into advancing diagnostic and therapeutic options to better assess prognostic factors and to improve treatment of cardiovascular atherosclerotic disease. A crucial part in reducing the mortality of patients suffering from atherosclerotic disease is an early reliable diagnosis and a prognostic risk assessment regarding cardiovascular incidents like infarction of heart muscle or brain tissue. Recent investigations have led to the hypothesis that composition and morphology of atherosclerotic plaques are good predictors for risk of cardiovascular incidents, rather than vascular stenosis alone [3-7].

A wide range of diagnostic methods like ultrasound, CT angiography and cardiovascular magnetic resonance (CMR) are currently in use to detect and characterize atherosclerotic plaques. Among these, plaque CMR is one of the most promising techniques. It is non-invasive and - with 3 T scanners becoming widely available - delivers excellent high resolution plaque images. More specifically, plaque morphology and components can reliably be identified and quantified, with excellent correlation to histopathology [3,8-14].

In vivo CMR plaque characterization at high detail has become a tool to investigate the relationship of certain plaque features with clinical symptoms. An increasing number of studies have addressed this issue, hypothesizing that certain plaque features like intraplaque hemorrhage, rupture of the fibrous cap, ulceration of plaque surface and formation of thrombi at the plaque surface are associated with cerebrovascular incidents [10,15-18].

However, most of these studies, except the study by De Marco Ota et al. 2010, were performed at 1.5 T MR scanners, scan times were relatively long (up to 45 minutes) and the number of excluded subjects was up to 32.5% [17-19], impeding its use in routine clinical studies. Furthermore, the time interval between symptom onset and CMR examination in most studies with up to 3 months was relatively long [15,17] and results have to be interpreted with caution, as plaque composition might have changed during this time. In addition, definition of symptomatic carotid disease varied wildly, e.g. many studies did not use brain MR to confirm ischemic events and TIA patients and patients with amaurosis fugax were also included.

The purpose of this prospective monocentric observational study was to determine if high-resolution 3 T magnetic resonance imaging with parallel imaging techniques can depict differences between symptomatic and asymptomatic carotid atherosclerotic plaques in acutely symptomatic macroangiopathic ischemic stroke patients referred from our interdisciplinary stroke center.

## Method

This study was in accordance with local regulatory legislation and approved by the institutional review board. All subjects gave written informed consent before CMR. The methods used in the study were in accordance with the ethical standards laid down in the Declarations of Helsinki.

### Patients

36 consecutive patients from the stroke unit of the Ludwig-Maximilians-University Hospital in Munich with unilateral acute symptomatic carotid disease were included in this study. Inclusion criteria were an ischemic stroke in the territory of the anterior or middle cerebral artery <10 days before black-blood carotid CMR and ≥30% carotid stenosis as determined by ultrasound according to the NASCET criteria [20]. Ischemic stroke was defined as an acute lesion on diffusion weighted brain MR images (DWI) and corresponding acute neurological deficit of more than 24 hours duration. The symptomatic artery was defined as being ipsilateral to the DWI lesion. Exclusion criteria were bilateral infarcts on brain MR, known contraindications against CMR, allergy to contrast material, impaired renal function defined as a glomerular filtration rate <30 ml/min, previous radiation therapy to head or neck and a surgical procedure within 24 h before black-blood carotid CMR.

All patients were subjected to the following investigations: laboratory analysis (including electrolytes, differential blood count, coagulation studies, and C-reactive protein), 12-lead electrocardiography, Holter monitoring, transthoracic echocardiography and/or transesophageal echocardiography (TEE), duplex sonography of the extracranial vessels, transcranial Doppler sonography, carotid black-blood CMR and brain MR (including DWI sequences). The aortic arch was evaluated for the presence of atherosclerotic plaques by using TEE; in addition, CT angiography (including the aortic arch) was available in the majority of patients. Additional tests (e.g., cerebrospinal fluid analysis, conventional angiography, screening for prothrombotic conditions) were performed if clinically indicated. Based on the results, patients were grouped according to the TOAST (Trial of Org 10172 in Acute Stroke Treatment) classification [21]. According to the TOAST classification, ischemic strokes of 24 patients with ≥50% stenosis were categorized into subtype I (large artery atherosclerosis) and 8 patients with ≥30% but <50% stenosis were categorized into subtype V (stroke of undetermined etiology). Demographics and cardiovascular risk factors like body mass index (BMI), present or past nicotine abuse, hypertension, diabetes mellitus, hypercholesterolemia and coronary heart disease (CHD) as well as family history of CHD were recorded.

### MR imaging protocol

All subjects underwent a carotid black-blood high resolution CMR at 3 T on a Siemens Verio Scanner (Siemens AG, Erlangen, Germany) using dedicated surface coils (Machnet, Elde, Netherlands) and a previously published multi-sequence protocol [22] (time-of-flight MR angiography (TOF), axial fat suppressed pre- and post- contrast black-blood T1-, PD- and T2- weighted sequences; best in-plane resolution 0.5 × 0.5 mm^2^). Each scan covered 30 mm (2 mm slice thickness × 15 matched images across the 5 sequences). This coverage is usually sufficient to image the complete carotid atherosclerotic plaque [23]. Parallel imaging based on the generalized auto calibrating partially parallel acquisition (GRAPPA) algorithm was used for all sequences with a parallel acquisition technique (PAT) acceleration factor of 2. Total imaging time was 17:43 minutes. Gadobutrol (Gadovist®, Bayer Schering, Leverkusen, Germany) of 0.1 mmol/kg (0.1 ml/kg) was given at a rate of 3 mL/s. Post-contrast T1w imaging was performed approximately 5 minutes after intravenous injection of the contrast agent.

### Image analysis

An image-quality rating (4-point scale, 1 = non diagnostic, 2 = poor, 3 = good, 4 = excellent) was assigned to all MR images. MR data was reviewed by two experienced radiologists with 11 (T.S.) and 4 years (J.M.G.) experience in carotid CMR who were blinded to the brain MR and to all clinical information of the patient. The initial review was done by J.M.G. and the final review by T.S. who was aware of the assessment of the initial review. In case of discrepancy, a consensus decision was made. Examinations with an image quality ≤ 2 were excluded from the study.

Atherosclerotic plaques in the carotid arteries (on both the ipsilateral and contralateral side) were recorded and classified according to the modified criteria of the American Heart Association [24]. Tissue components (necrotic core, calcification, hemorrhage, loose matrix) and type and location of hemorrhage were identified and quantified based on previously published criteria [3,8,25,26]. For definition of a complicated AHA-LT6 plaque, at least one of the following three criteria was required: Fibrous cap rupture, intraplaque hemorrhage, juxtaluminal hemorrhage/mural thrombus. Area measurements of the lumen, outer wall, and tissue components were obtained using the custom-designed image analysis tool CASCADE (University of Washington, Seattle, US) [27]. The “total vessel area” included the lumen and wall areas. The wall area for each location was calculated as the difference between the total vessel and lumen areas. The normalized wall index (NWI) was calculated by dividing the wall area by the total vessel area.

### Statistical analysis

Each patient contributed only one set of observations (one study of the symptomatic artery, one study of the asymptomatic arteries) to the data set for analysis. Area measurements for each artery were calculated as the minimum and maximum areas for each artery separately. Categorical variables are presented as relative and absolute frequencies, while continuous variables are presented as mean and standard deviation. Wilcoxon’s signed rank test was used to test differences for continuous variables, and the McNemar test was used to determine differences between categorical variables. We also derived the relative risk (odds ratio, OR) associated with AHA-LT6, intraplaque hemorrhage, juxtaluminal hemorrhage/thrombus, and a ruptured fibrous cap as determined by CMR for the presence of symptoms using conditional logistic regression analysis taking into account the clustered data structure per subject. A p-value <0.05 was considered statistically significant. Analyses were carried out using SPSS 16.0 for Windows (International Business Machines Corp., New York, USA) and SAS 9.2 (Cary, NC).

## Results

### Image quality

In 34 out of 36 patients (94.4%) image quality of CMR examinations was sufficient (≥ 2) to be included in the study with a mean image quality rating of 3.8. All data in this evaluation is based on these 34 patients. Two patients had to be excluded due to severe motion artifacts.

### Demographics and clinical data

**Figure 1:**
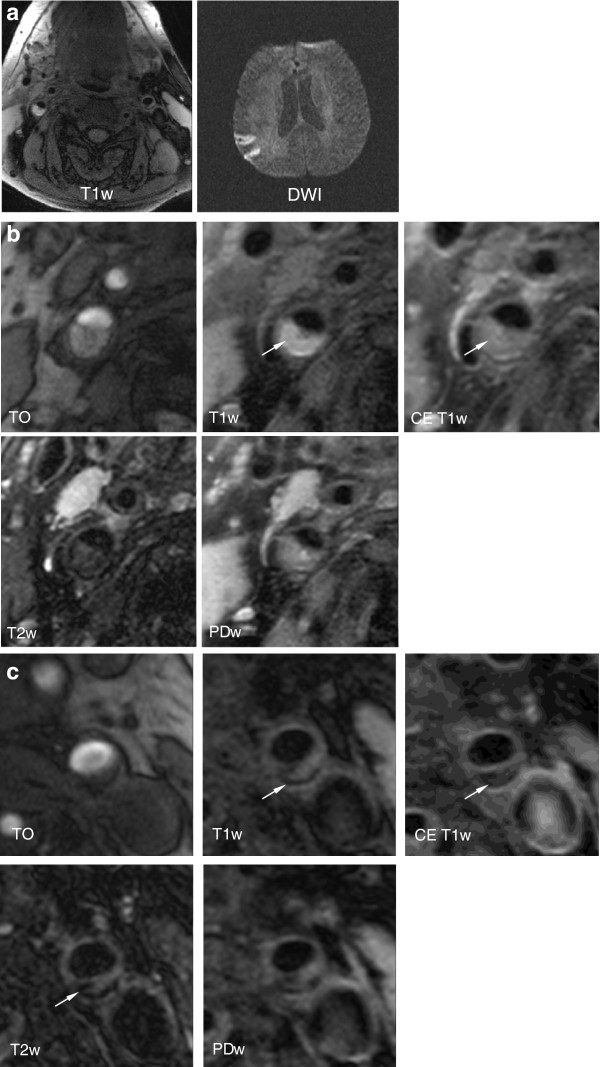
**Imaging example.** Figure 1 shows various CMR-images of a 66 years old patient who suffered an infarction 4 days before the CMR scan. **a**. T1w and DWI MR-images of the patient. Axial T1w images with fat suppression demonstrate atherosclerotic lesions in both internal carotid arteries (ICA). The lesion in the right ICA, ipsilateral to the symptoms, is hyperintense, consistent with intraplaque hemorrhage. The lesion in the left ICA is hypo- to isointense, consistent with a fibrous and calcified lesion. Axial DWI images show a diffusion restriction in the posterior part of the right middle cerebral artery, consistent with an acute brain infarction. **b**. Axial multi-sequence CMR images of the same patient demonstrating the complicated AHA lesion type VI plaque ipsilateral to the stroke in the right ICA. The arrow points to a region which is hyperintense on T1w and TOF images and hypointense on PDw and T2w images, consistent with type I hemorrhage into a large necrotic core. The dark band on TOF images is disrupted, the hyperintense area on TOF is located juxtaluminally and the fibrous cap cannot be visualized in the contrast enhanced T1w images, indicating rupture of the fibrous cap (ICA = Internal carotid artery, TOF = Time-of-Flight). **c**. Axial multi-sequence CMR images of the same patient demonstrating the uncomplicated AHA lesion type VII plaque contralateral to the stroke in the left ICA. The arrow points to a region which is hypointense on all images, consistent with a calcification, surrounding an area which is isointense to fatty tissue in T1w, T2w and PDw images and shows no contrast enhancement, consistent with a necrotic lipid core. The contrast enhancing fibrous cap separating the plaque from the lumen appears intact. (ICA = Internal carotid artery, TOF = Time-of-Flight).

Clinical data of the 34 included patients are displayed in Table 1. An overview of the imaging results is given in Table 2, ORs with 95% confidence intervals are summarized in Table 3. An imaging example is given in Figure 1.

### Prevalence of plaque features ipsilateral and contralateral to ischemic stroke

**Figure 2:**
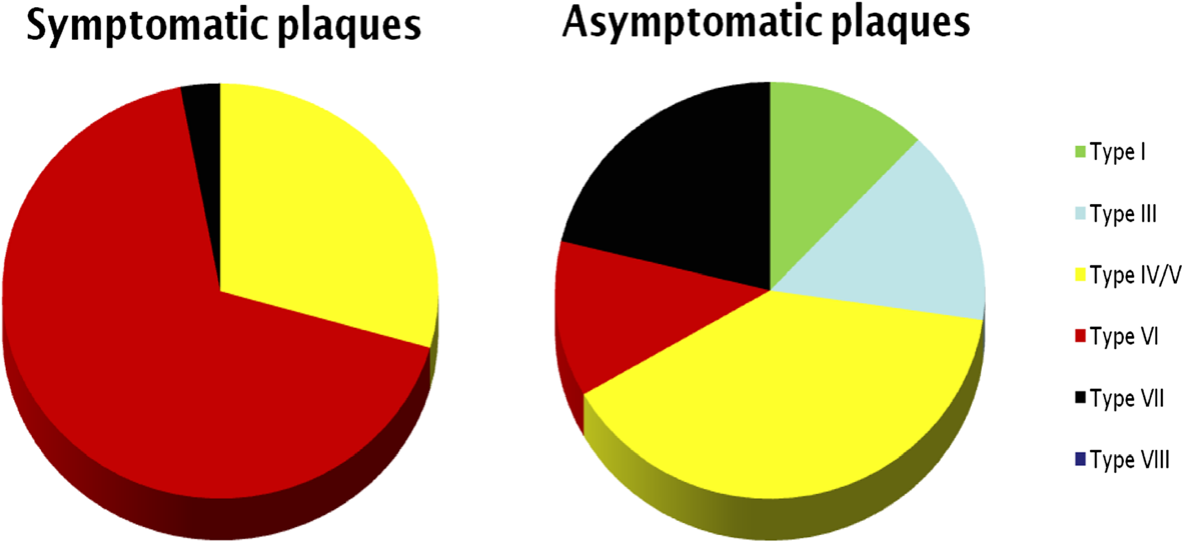
**Distribution of AHA lesion type.** Figure 2 visualizes the distribution of AHA lesion types in the asymptomatic and the symptomatic groups. Note the higher prevalence of AHA lesion type 6 plaques in the symptomatic group and of types 3 and 7 in the asymptomatic group.

In the qualitative analysis, symptomatic plaques had a higher prevalence of AHA-LT6 compared to asymptomatic plaques (67.6% vs. 11.8%; p < 0.001; OR = 12.5), while in asymptomatic plaques AHA-LT3 and 7 were more frequent (14.7% vs. 0% and 20.6% vs. 2.9%; p < 0.05, respectively) [Figure 2]. A ruptured fibrous cap was found more frequently in the symptomatic than in the asymptomatic group (44.1% vs. 2.9%; p < 0.001; OR = 15.0). Prevalence of haemorrhage (intraplaque haemorrhage and juxtaluminal haemorrhage/thrombus combined) was significantly higher in the symptomatic group (67.7% vs. 11.8%; p < 0.001). Several patients had a combination of intraplaque hemorrhage and juxtaluminal hemorrhage/thrombus. More specifically, prevalence of intraplaque hemorrhage was 58.6% in the symptomatic group and 11.8% in the asymptomatic group (p < 0.01; OR = 3.8). Juxtaluminal hemorrhage

**Table 1:** Clinical data

**Parameter**	**Value [n = 34 patients]**
Age ± 1 SD [years]	70 ± 9.3
Male [%] (n)	71% (24)
Mean Body Mass Index ± 1 SD	25.8
**Risk factors**	
Hypercholesterolemia/Hyperlipidemia [%] (n)	53% (18)
Arterial hypertension [%] (n)	62% (21)
Active nicotine abuse [%] (n)	26% (9)
Former nicotine abuse [%] (n)	35% (12)
Diabetes mellitus [%] (n)	18% (6)
CHD or CVD [%] (n)	18% (6)
Family history of CHD or CVD [%] (n)	29% (10)

* McNemar Test; SD = Standard Deviation; CHD = Coronary Heart Disease; CVD = Cerebrovascular Disease (TIA or Stroke).

**Table 2:** CMR data

	**Symptomatic plaques (n = 34 arteries)**	**Asymptomatic plaques (n = 34 arteries)**	p-value*
Mean lumen area [mm^2^]	24.9 ± 11.1	26.7 ± 11.3	n.s.
Mean wall area [mm^2^]	43.0 ± 13.0	35.9 ± 10.2	0.04
Normalized wall index	0.66 ± 0.1	0.59 ± 0.1	n.s.
Mean total vessel area [mm^2^]	67.9 ± 21.4	62.5 ± 17.7	n.s.
**Area measurements of plaque components**			
Max. necrotic core, [mm^2^]	14.1 ± 9.7	5.5 ± 7.5	0.001
Max. intraplaque hemorrhage, [mm^2^]	8.0 ± 9,3	1.3 ± 7.5	0.007
Max. loose matrix, [mm^2^]	0.7 ± 2.1	1.0 ± 3.2	n.s.
Max. calcification, [mm^2^]	2.2 ± 3.3	2.4 ± 3.8	n.s.
**Plaque components as percentage of the vessel wall (in%)**			
Max. necrotic core, [%]	25.9 ± 14.6	11.2 ± 13.3	0.001
Max. intraplaque hemorrhage, [%]	14.3 ± 19.9	2.2 ± 6.7	0.003
Max. loose matrix, [%]	1.6 ± 4.1	1.7 ± 5.0	n.s.
Max. Calcification, [%]	4.1 ± 5.5	5.1 ± 6.1	n.s.
**Prevalence of plaque features**			
Juxtaluminal hemorrhage/thrombus [%] (n)	26.5% (9)	0	<0.01
Intraplaque hemorrhage [%] (n)	58.6% (20)	11.8% (4)	<0.01
Any hemorrhage [%] (n)	67.6% (23)	11.8% (4)	<0.001
Ruptured fibrous cap [%] (n)	44.1% (15)	2.9% (1)	<0.001
Necrotic core [%] (n)	94.1% (32)	55.8% (19)	<0.001
Calcification [%] (n)	52.9% (18)	55.8% (19)	n.s.
**American heart association (AHA) lesion type**			
Type I [%] (n)	0	11.8% (4)	n.s.
Type III [%] (n)	0	14.7% (5)	0.02
Type IV/V [%] (n)	29.4% (10)	41.2% (14)	n.s.
Type VI [%] (n)	67.6% (23)	11.8% (4)	<0.001
Type VII [%] (n)	2.9% (1)	20.6% (7)	0.03
Type VIII [%] (n)	0	0	n.s.

* McNemar test or Wilcoxon Signed Rank test; Values are given as mean ± *Standard Deviation.*

 and/or thrombus was detected significantly more frequently in the symptomatic group (26.5% vs. 0%; p < 0.01; OR = 7.3).

Plaques showed a necrotic core more frequently in the symptomatic than in the asymptomatic group (94.1% vs. 55.8%; p < 0.001). In contrast, prevalence of intraplaque calcification was not significantly different between the

**Table 3:** Odds ratios of qualitative CMR variables for the presence of symptoms

**Variable**	**Odds ratio (95% CI)**
Intraplaque hemorrhage	3.75 (1.2 – 11.3)
Juxtaluminal hemorrhage/thrombus	7.3 (2.2 – 24.5)
Ruptured fibrous cap	15.0 (2.0 – 113.6)
AHA lesion type VI	12.5 (3.0 – 52.8)

*CI* Confidence Interval, *AHA* American Heart Association.

 symptomatic and asymptomatic group (52.9% vs. 55.8%; p = 0.81).

### Quantitative plaque characteristics ipsilateral and contralateral to ischemic stroke

**Figure 3:**
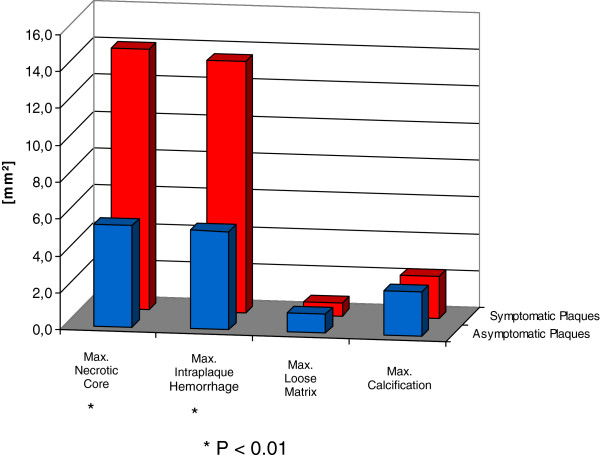
**Quantitative analysis of plaque components.** Figure 3 shows the absolute quantitative distribution of different plaque features in mm^2^ on cross sectional images for symptomatic and asymptomatic plaques in comparison. Symptomatic plaques showed a greater maximum cross sectional area for lipid rich necrotic cores and hemorrhage while no differences were found for loose matrix and calcification areas.

In the quantitative analysis [Figure 3], maximum necrotic core and intraplaque hemorrhage area were greater in symptomatic than in asymptomatic plaques (14.1 mm^2^ vs. 5.5 mm^2^ and 8.0 mm^2^ vs. 1.3 mm^2^; p < 0.01, respectively), while mean vessel and lumen areas did not significantly differ between both groups. Mean wall area was greater in the symptomatic group (43.0 vs. 35.9 mm^2^; p = 0.04).

### Effect of the TOAST classification and demographics on qualitative and quantitative plaque characteristics

No significant differences in the prevalence of complicated plaques was found between the 8 patients with

 30-49% stenosis (classified as stroke with undetermined etiology according to TOAST) and the 24 patients with ≥50% stenosis (50% vs. 71%, p = 0.4). No other significant differences were found.

## Discussion

Findings of our study demonstrated that 3 T high resolution in vivo CMR using parallel imaging and dedicated surface coils is a reliable, fast and robust technique, which allows to non-invasively depict significant differences between symptomatic and asymptomatic carotid plaques in patients with acute ischemic stroke.

In our study we aimed to eliminate limitations of previous examinations and to determine if high-resolution 3 T magnetic resonance imaging using Parallel Imaging techniques can depict differences between symptomatic and asymptomatic carotid atherosclerotic plaques in patients with acute ischemic stroke. Existing studies, for example, were mainly performed on 1.5 T CMR scanners [17-19] and no Parallel Imaging techniques were used [15,17-19,28]. While in most studies the number of excluded subjects was relatively large with 15% [17], 19% [18,28] and 32.5% [19], only 5.6% (2 out of 34) of our patients had to be excluded due to insufficient image quality in our study. A 3 T-CMR study of Demarco et al. [15] excluded 7.2% of patients, but included only 13 symptomatic patients and was not correlated with diffusion weighted MR. Also, the interval between symptom onset and CMR was longer in some studies with up to eight weeks [28] or up to four months

[15,17]. This is the first study to examine a relevant number of patients suffering from acute unilateral ischemic stroke using high resolution 3 T in vivo plaque imaging with Parallel Imaging techniques and dedicated surface coils. The MR imaging protocol used in our study as described by Saam et al. [22] took only 17:43 minutes and was thus considerably shorter than some of the protocols used in other studies with around 22 minutes reported by Demarco et al. [15] and approximately 40 minutes in the study by Boussels [19]. The shorter acquisition time in combination with the higher field strength might be the main reason for the relatively low exclusion rate observed in our study. One potential advantage of our imaging platform which was not explored in our study is the potential to perform the brain MR and the carotid CMR in the same imaging session without the necessity to change the coils. This approach could help to establish carotid plaque CMR as a useful clinical tool in patients with acute ischemic stroke.

High resolution in vivo 3 T black blood CMR using dedicated surface coils and Parallel Imaging techniques can depict differences between symptomatic and asymptomatic plaques and thus differentiate between symptomatic and asymptomatic lesions as suggested by previous studies. The most potent single predictor for the symptomatic side in our study was a ruptured fibrous cap with an OR of 15.0 (95% CI = 2.0 – 113.6).  An AHA lesion type VI and juxtaluminal thrombus were slightly inferior predictors with ORs of 12.5 (95% CI = 3.0 – 52.8) and 7.3 (95% CI = 2.2 – 24.5), respectively, while intraplaque hemorrhage had the lowest OR (3.75; 95% CI = 1.2–11.3). Therefore, it seems to be important to apply MR sequences, which can detect a rupture of the fibrous cap as well as intraplaque hemorrhage and juxtaluminal hemorrhage/thrombus in order to assess plaque vulnerability. Findings of our study are consistent with a study by Parmar et al. [18], which included 78 patients with 40 TIAs/strokes.

Demarco et al. [15] recently published a study at 3 T with 13 symptomatic and 77 asymptomatic subjects. The plaque features that showed the highest association with symptoms differed between subjects with moderate and severe stenosis. In patients with severe carotid stenosis ulceration on MRA demonstrated the strongest association with symptoms with an OR of 10.5, while in patients with moderate stenosis presence of intraplaque hemorrhage showed the strongest association with symptoms with an OR of 6.0. Interestingly, intraplaque hemorrhage was not significantly associated with symptoms in patients with severe carotid stenosis (OR = 1.3).

In our study we found a slightly higher prevalence of AHA lesion Type VI in plaques with carotid stenosis ≥50% compared to plaques with 30-49% stenosis. Findings of a previously published study with 192 patients showed an increasing prevalence of AHA lesion type VI with increasing degree of stenosis in asymptomatic subjects [29]. While our results point in the same direction, they do not fully confirm these findings, which may be attributed to the smaller number of patients in our study. We did not find a higher prevalence of AHA lesion type VI in male compared to female patients. This was proposed in a previous report by Ota et al. (Stroke 2010) with 131 asymptomatic patients (men, 67; women, 64) with ≥50% carotid stenosis on duplex ultrasound showing a higher prevalence of a ruptured fibrous cap (48% versus 17%, adjusted OR = 4.41, p < 0.01) and lipid-rich necrotic core (73% versus 50%, adjusted OR = 3.66, p = 0.01) in men as well as a trend for higher prevalence of hemorrhage (33% versus, 17%, adjusted OR = 2.15, p = 0.07) in men. However, this study included only asymptomatic patients and our study suggests that these findings cannot be applied in symptomatic populations.

### Limitations

Although we found highly significant differences between symptomatic and asymptomatic plaques, there was a significant overlap of some of these plaque features between symptomatic and asymptomatic plaques, suggesting that a complete separation of symptomatic and asymptomatic subjects is not feasible using a single CMR variable. This limitation might be overcome by using a scoring system based on a number of variables, such as previously proposed by Gury-Paquet et al.[28]or Underhill et al. [29].

## Conclusion

Our results confirm that 3 T high resolution in vivo CMR using parallel imaging and dedicated surface coils is a potent and fast tool in the assessment of carotid atherosclerotic disease and could be easily implemented into the clinical routine with a scan time of <25 minutes. The imaging procedure is robust, yielding an image quality that is sufficient for detailed plaque assessment in the vast majority of cases. In our study, a ruptured fibrous cap was the dominant outcome-associated observation achieving significance with an OR of 15.0. Prevalence of a complicated AHA lesion type VI, which is characterized by hemorrhage, thrombus or a ruptured fibrous cap, was a comparably potent predictor for the symptomatic side with an OR of 12.5. In the future, prospective studies will have to determine the predictive value of AHA lesion type VI in general and a ruptured fibrous cap in particular on cerebrovascular symptoms. One currently on-going study started in February 2011 is the CAPIAS trial (Carotid Plaque Imaging in Acute Ischemic Stroke; ClinicalTrials.gov Identifier: NCT01284933), which evaluates the prevalence and effects of AHA lesion type VI in patients with acute ischemic stroke and <70% stenosis. Findings of the CAPIAS study will provide valuable insights into stroke mechanisms and could be the basis for planning targeted interventional studies, which could focus on a combination of plaque morphology and the degree of luminal stenosis.

## Competing interests

The authors declare that they have no competing interests.

## Authors’ contributions

JMG participated in data acquisition, statistical evaluation, drafted and revised the manuscript. AS participated in patient and data acquisition and revision of the manuscript. CF participated in designing the study and evaluating the data. FB participated in evaluating and interpreting the data. CCC participated in patient and data acquisition. CY participated in evaluating the data and revising the manuscript. CF participated in evaluating the data. MD participated in designing the study, evaluating the data and revising the manuscript. MFR participated in designing the study and revising the manuscript. KN participated in designing the study and revising the manuscript. TS participated in designing the study, acquiring and evaluating the data, and revising the manuscript. All authors read and approved the final manuscript.
